# Outcomes in reported penicillin allergic mothers and neonates requiring Group B streptococcal prophylaxis: a retrospective observational cohort study

**DOI:** 10.1186/s12887-021-02797-8

**Published:** 2021-07-27

**Authors:** Justin Kirven, David Beddow, Love Patel, Claire Smith, Katherine S. Booker, Barite Dawud, Catherine A. St. Hill

**Affiliations:** 1 Department of Internal Medicine, Abbott Northwestern Hospital, Allina Health, 800 E 28th St, Minneapolis, MN 55407 USA; 2 Department of Internal Medicine, Mercy Hospital, Allina Health, Minneapolis, MN USA; 3 Care Delivery Research, Allina Health, Minneapolis, MN USA

**Keywords:** Hypersensitivity, Infant, Newborn, Penicillins, Pregnancy, Streptococcus

## Abstract

**Background:**

Infectious morbidity and mortality in the first week of life is commonly caused by early-onset neonatal Group B streptococcus (GBS) disease. This infection is spread from GBS positive mothers to neonates by vertical transmission during delivery and results in serious illness for newborns. Intrapartum prophylactic antibiotics have decreased the incidence of early-onset neonatal GBS disease by 80%. Patients labeled with a penicillin allergy (PcnA) alternatively receive either vancomycin or clindamycin but effectiveness is controversial. We evaluated the influence of a reported PcnA label versus no PcnA label on inpatient maternal and neonatal outcomes.

**Methods:**

Our goal was to examine the relationship between a PcnA label, maternal and neonatal outcomes, and hospital costs. We collected retrospective data with institutional IRB approval from 2016 – 2018 for hospitalized patients who were GBS positive, pregnant at time of admission, ≥ 18 years of age, received antibiotic prophylaxis for GBS, were labeled as PcnA or non-PcnA, and completed a vaginal delivery. Patient characteristics and maternal/neonatal outcomes were examined. Statistical tests included calculations of means, medians, proportions, Mann–Whitney, two-sample t-tests, Chi-squared or Fisher’s Exact tests, and generalized linear and logistic regression models. Significance was set at *p* < 0.05.

**Results:**

Most PcnA patients were white, older, had a higher median body mass index and mean heart rate, and a greater proportion used tobacco than non-PcnA patients. In regression analyses, PcnA hospitalized patients received a shorter duration of antibiotic treatment than non-PcnA patients [incidence rate ratio (IRR): 0.45, 95% CI: 0.38–0.53]. PcnA patients were also more likely to have their baby’s hospital LOS be > 48 h [adjusted odds ratio (AOR): 1.35, 95% CI: 1.07–1.69] even though the PcnA mothers’ LOS was not different from non-PcnA mothers. Cost of care, mortality, intensive care, median parity, mean gravidity, and miscarriage were similar between the groups.

**Conclusions:**

In hospitalized obstetric patients, a PcnA label was associated with a shorter maternal course of antibiotic treatment and a longer neonatal LOS. Further prospective studies are needed to clarify the underlying reasons for these outcomes.

## Introduction

Group B streptococcus (GBS) is the most common etiology of infectious morbidity and mortality in the first week of neonatal life worldwide [1]. This infection is spread to neonates by vertical transmission during delivery from mothers who are infected or colonized with the bacteria. For well over a decade, it has been standard of care to universally screen women in their third trimester of pregnancy for GBS by culture-based screening and to administer prophylactic intrapartum antibiotics to women who test positive or have specific risk factors for GBS [2, 3].

Intrapartum antibiotic prophylaxis (IAP) for selected populations has reduced the incidence of early-onset neonatal GBS disease by an estimated 80% as compared to the pre-IAP era [4]. Penicillin G and ampicillin are the preferred IAP agents due to the high susceptibility of GBS to these drugs and the rapidly achieved effective concentrations in cord blood and amniotic fluid [5]. Cefazolin is a similarly effective alternative for those with PcnA at low risk for anaphylaxis [3]. In contrast, mothers who carry a label of penicillin allergy (PcnA) often receive alternative prophylaxis regimens due to concern for a severe allergic reaction, despite the fact that many patients who carry a PcnA label are at low risk for a serious Ig-E mediated allergic reaction [6]. Alternative antibiotics used in PcnA patients such as vancomycin and clindamycin have disadvantages when compared to first-line GBS IAP. GBS resistance to clindamycin is estimated as high as 40% and clindamycin takes longer to reach effective concentrations in amniotic fluid [7, 8]. Vancomycin is associated with the spread of drug-resistant organisms and is a target for antibiotic stewardship internationally [9].

Despite a high frequency of PcnA labeling in obstetric patients, there is a dearth of data to describe the demographic and clinical characteristics of these patients. Moreover, there is little data to compare the hospital course of neonates and mothers with a maternal PcnA label. We hypothesized that a PCNa label was associated with increased hospital costs and adverse maternal and neonatal outcomes.

## Methods

### Study design, population, and data collection

The goal of this study was to compare demographics and clinical outcomes for mothers infected with GBS and their neonates in order to determine the impact of a PcnA label on maternal and neonatal care. The Institutional Review Board (IRB) of Allina Health system approved this project as an exempt study # 1,380,327 according to federal regulation 45 CFR 46.104(d) and the need for informed consent was waived. For this retrospective observational cohort study, inclusion criteria were hospitalized patients from 2016–2018 within the Allina Health system, who were GBS positive, pregnant at time of admission, ≥ 18 years of age, received antibiotic prophylaxis for GBS, were labeled as PcnA or non-PcnA, and completed a vaginal delivery. Hospitalized patients < 18 years of age were excluded.

### Outcomes

We compared outcomes for hospitalized mothers and their neonates with a penicillin allergy (PcnA) label. The primary maternal outcome was length of stay (LOS). Secondary outcomes were total cost of care, days of antibiotic use, peripherally inserted central catheter (PICC) use, intensive care unit (ICU) admission either pregnant or not, LACE score (readmission risk scoring), and 30-day hospital or emergency department (ED) readmission. The primary neonatal outcome was LOS > 48 h. Secondary neonatal outcomes were transfer to a higher level of care, and fetal death or miscarriage.

### Statistical analysis

As appropriate, descriptive methods, including means and standard deviations, medians and interquartile ranges Q1-Q3, frequencies and proportions, were used to describe patient characteristics and outcomes for the PcnA and non-PcnA labeled groups. Comparisons between the PcnA and non-PcnA labeled groups were conducted using two-sample independent t-tests, Mann–Whitney U tests, chi-square tests, and Fisher’s exact tests.

Adjusted analyses were conducted with the primary maternal and newborn outcomes, hospital LOS and baby LOS > 48 h. Adjusted analyses were also conducted with two secondary maternal outcomes of high clinical interest, total cost of care and days on antibiotics. Covariates for the models, body mass index (BMI) > 35, age > 35 years, tobacco use, and pregnancy type, were selected based on clinical importance. Adjusted odds ratios and their respective 95% confidence intervals (CI) were calculated for baby LOS > 48 h using logistic regression. The assumptions of the regression models were met, diagnostic plots were assessed, and no violations, including influential observations and multicollinearity, were found.

Differences between the PcnA and non-PcnA labeled groups in hospital LOS and duration of antibiotics were tested using negative binomial regression. Negative binomial regression is a generalization of Poisson regression and can be used for modeling over-dispersed (conditional variance is greater than the conditional mean) count dependent variables. For maternal hospital LOS and days of antibiotic use data, the possible values were non-negative integer values. The variances within the two categories, PcnA labeled and non-PcnA labeled, exceeded the within-category means.

Differences in total cost of care were assessed using gamma regression. Gamma regression can be used for modeling right skewed positive continuous outcome variables. Analysis of total cost of care during a hospital stay data is an example of always positive and highly right skewed data that can be a good fit for gamma modeling.

Two-tailed *p*-values were computed for all tests, with a *p* < 0.05 value considered significant. All statistical analyses were conducted using R software version 3.6.1 (The R Foundation for Statistical Computing, Vienna, Austria.)

## Results

We analyzed characteristics and outcomes of obstetric patients who were PcnA labeled or not labeled. Retrospective data were extracted from medical records of 4,387 hospitalized GBS positive pregnant patients who had a vaginal delivery. Of these, 506 (11.5%) were PcnA labeled and 3881 (88.5%) were not (Table 1).

**Table 1:** Characteristics of obstetric patients with a PcnA label versus no PcnA label

	**PcnA label**	**No PcnA label**	***p*-value**
Number of patients, n (%)	506 (11.5%)	3881 (88.5%)	
Height (cm), median (IQR)	166.4 (162.6—170.2)	165.1 (160.0—168.9)	< 0.001*
Weight (kg), median (IQR)	84.4 (76.2—97.4)	82.1 (72.6—93.6)	< 0.001*
BMI, median (IQR)	30.7 (27.6—35.5)	30.4 (27.1—34.4)	0.03*
BMI > 35, n (%)	122 (24.1)	729 (18.8)	0.01*
Heart rate, mean (SD)	84.6 (10.8)	83.2 (10.9)	0.007*
Age in years, mean (SD)	30.1 (5.3)	29.6 (5.4)	0.03*
Age > 35 years, n (%)	83 (16.4)	544 (14.0)	0.15
Race, n (%)			< 0.001*
White	429 (86.0%)	2571 (68.3%)	
Non-White	70 (14.0%	1191 (31.7%)	
Latino/Hispanic, n (%)	25 (5.0%)	228 (6.0%)	0.39
Insurance, public, n (%)	159 (31.4%)	1540 (39.7%)	< 0.001*
Tobacco use, n (%)			< 0.001*
Yes	178 (35.6%)	981 (25.6%)	
Admission type, n (%)			0.70
Elective	110 (21.7%)	799 (20.6%)	
Emergency	0 (0.0%)	3 (0.1%)	
Urgent	396 (78.3%)	3079 (79.3%)	
Antibiotic type, n (%)	*n* = 370	*n* = 155	* < 0.001
Penicillins	0 (0.0%)	34 (21.9%)	
Cephalosporins	205 (55.4%)	83 (53.5%)	
^a^Other	165 (44.5%)	38 (24.5%)	
Pregnancy type, n (%)			0.40
Singleton	500 (97.8%)	3845 (98.2%)	
Twin	12 (2.3%)	69 (1.8%)	

^*^Statistical significance was set at *p* < 0.05. *BMI* body mass index, *IQR* interquartile range, *PcnA* penicillin allergy, *SD* standard deviation. ^a^Other: non-penicillin and non-cephalosporin antibiotics

Compared to non-PcnA labeled obstetric patients, a greater proportion of patients with a PcnA label were white (86.0% vs. 68.3%), older (mean age of 30.1 years vs. 29.6 years old), had a higher median body mass index (BMI of 30.7 vs. 30.4), and had a higher mean heart rate (Table 1). A higher proportion of PcnA labeled patients used tobacco (35.6% vs. 25.6% for non-PcnA) and a lower proportion used public insurance (31.4% for PcnA vs. 39.7% for non-PcnA). The use of different types of antibiotics (penicillins, cephalosporins, and other types, *p* < 0.01) differed for PcnA labeled compared to non-PcnA patients.

Past medical history for patients with a PcnA label and non-PcnA labeled patients was not different for cancer, heart failure, chronic obstructive pulmonary disease, chronic kidney disease, diabetes mellitus, hypertension, sleep apnea, coronary artery disease, stroke, liver disease, intracranial hemorrhage, craniotomy, pulmonary embolism, deep vein thrombosis, thrombophilia, and pulmonary fibrosis. Differences were not observed between the two groups for severity of illness (SOI) as measured by the APR-DRG SOI classification scoring, risk of readmission (mean LACE score), risk of mortality, inpatient or 30-day post-discharge mortality, ventilator use, inpatient or 6-month post discharge *Clostridioides difficile* infection, or CLABSI including PICC use, or discharge to home (data not shown). Median parity, and mean gravidity were not different between the groups. There were no differences between the PcnA labeled group and non-PcnA labeled mothers for gestational age at delivery and birth weight of the neonate (data not shown). Type of admission and type of pregnancy were also similar for PcnA and non-PcnA labeled patients (Table1).

We evaluated primary unadjusted outcomes for mothers and newborns with and without a PcnA label. When the outcomes were not controlled for the influence of other variables examined in the analyses, the median hospital LOS was longer for PcnA labeled than for non PcnA patients (2.13 days vs. 2.06 days, *p* = 0.01, Table 2). Four patients in the non-PcnA labeled group compared to none of the patients with a PcnA label required an ICU stay. Deaths did not occur in both groups during the hospitalization and for up to 30 days post discharge. In the unadjusted outcomes, median total cost of care during the hospital stay was higher for PcnA labeled patients ($6,796 vs. $6,444 for non-PcnA, *p* = 0.004) and 19 (3.8%) of these patients required a PICC whereas non-PcnA labeled patients did not (*p* < 0.001). Differences existed in the mean number of days on antibiotics (1.3 days for PcnA labeled vs. 2.7 days for non-PcnA, *p* < 0.01) for PcnA labeled compared to non-PcnA patients.

**Table 2:** Unadjusted outcomes of obstetric patients with a PcnA label versus patients without a PcnA label

	**PcnA label**	**No PcnA label**	***p*-value**
Number of patients, n (%)	506 (11.5%)	3881 (88.5%)	
**Primary Maternal Outcomes**			
Hospital LOS in days, median (IQR)	2.13 (1.74—2.44)	2.06 (1.69—2.36)	*0.01
**Secondary Maternal Outcomes**			
Total cost of care, dollars, median (IQR)	6796 (5344—8839)	6444 (5023—8489)	*0.004
Days of antibiotic use, mean (SD)	1.3 (0.6)	2.7 (4.5)	* < 0.001
30-day readmission, ED and IP, n (%)	20 (4.0%)	205 (5.3%)	0.26
PICC line use, n (%)	19 (3.8%)	0 (0.0%)	* < 0.001
ICU admission, n (%)	0 (0.0%)	4 (0.1%)	> 0.99
Pregnancy at ICU admission, n (%)	0 (0.0%)	1 (25%)	NA
**Primary Neonatal Outcomes**			
Baby LOS > 48 h, yes, n (%)	133 (26.0%)	831 (21.2%)	*0.01
**Secondary Neonatal Outcomes**			
Level 2 nursery, n (%)	62 (12.1%)	494 (12.6%)	0.74
Birth outcome, n (%)			> 0.99
Live birth	512 (100%)	3908 (99.9%)	
Fetal death or miscarriage	0 (0.0%)	6 (0.2%)	

^*^Statistical significance was set at *p* < 0.05. *ED* emergency department, *ICU* intensive care unit, *IP* inpatient, *IQR* interquartile range, *LOS* length of stay, *PcnA* penicillin allergy, *PICC* peripherally inserted central catheter, *SD* standard deviation

In the unadjusted outcomes, neonates of PcnA labeled mothers were significantly more likely to have a LOS of > 48 h than neonates of non-PcnA labeled mothers (26.0% vs 21.2%) (Table 2). There were no differences in neonates born to PcnA or non-PcnA labeled mothers for transfer to a higher level of care such as a level 2 nursery, fetal death, or miscarriage. Importantly, there were no differences between the PcnA labeled group and non-PcnA labeled mothers for return to the ED or readmission to the hospital (Table 2).

We performed regression analyses on the primary maternal outcome hospital LOS and the primary neonatal outcome baby LOS > 48 h. We also performed regression analyses on the secondary outcomes total cost of care and days of antibiotic use. For these analyses, PcnA status was the independent variable of interest and the primary and secondary outcomes were adjusted for the influences of advanced maternal age (age > 35 years), BMI > 35, tobacco use, and twin delivery.

PcnA labeled patients received a shorter duration of antibiotic treatment than non-PcnA patients [incidence rate ratio (IRR): 0.45, 95% CI: 0.38–0.53] (Table 3). PcnA labeled patients were also more likely to have their baby’s hospital LOS be > 48 h [adjusted odds ratio (AOR): 1.35, 95% CI: 1.07–1.69] (Table 4). The results of the regression analysis did not show a significant difference in hospital length of stay for mothers [incidence rate ratio (IRR): 1.00, 95% CI: 0.93–1.08] (Table 5) and total cost of care between PcnA labeled and non-PcnA patients (data not shown).

**Table 3:** Negative binomial regression of days on antibiotics for hospitalized patients

Predictors	Incidence Rate Ratios	95% CI	*p*-value
PcnA label	0.45	0.38 – 0.53	< 0.001*
BMI > 35	1.10	0.90 – 1.34	0.355
Age > 35 years	1.04	0.83 – 1.30	0.749
Twin pregnancy	1.38	0.94 – 2.01	0.099
Tobacco use	0.83	0.68 – 1.00	0.055

^*^Statistical significance was set at *p* < 0.05, *n* = 440,440. *BMI* body mass index, *PcnA* penicillin allergy. Days on antibiotics were adjusted for BMI, age, twin pregnancy, and tobacco use in the regression analyses

**Table 4:** Logistic regression of neonatal hospital length of stay > 48 h

Predictors	Adjusted Odds Ratios	95% CI	*p*-value
PcnA label	1.35	1.07 – 1.69	0.011*
BMI > 35	1.02	0.84 – 1.23	0.826
Age > 35 years	1.06	0.85 – 1.32	0.609
Twin pregnancy	1.33	0.76 – 2.21	0.298
Tobacco use	0.95	0.79 – 1.14	0.603

^*^Statistical significance was set at *p* < 0.05, *n* = 3,676. *BMI* body mass index, *PcnA* penicillin allergy. Neonatal hospital length of stay > 48 h was adjusted for BMI, age, twin pregnancy, and tobacco use in the regression analyses

**Table 5:** Negative binomial regression of maternal hospital length of stay

Predictors	Incidence Rate Ratios	95% CI	*p*-value
PcnA label	1.00	0.93 – 1.08	0.935
BMI > 35	1.05	0.99 – 1.11	0.08
Age > 35 years	1.00	0.94 – 1.07	0.967
Twin pregnancy	2.09	1.78 – 2.45	< 0.001*
Tobacco use	1.00	0.94 – 1.05	0.862

^*^Statistical significance was set at *p* < 0.05, *n* = 3630. *BMI* body mass index, *PcnA* penicillin allergy. Maternal hospital length of stay was adjusted for BMI, age, twin pregnancy, and tobacco use in the regression analyses

## Discussion

Penicillin allergy is common in the US, with approximately 10% of the population reporting an allergy to penicillin [10]. However, the incidence of clinically significant IgE-mediated or T lymphocyte-mediated hypersensitivity is uncommon, and IgE sensitivity can wane over time [10]. In the general medical population, the label of PcnA has been associated with a variety of adverse outcomes, including the use of suboptimal or broader spectrum antibiotics [11], increased risk of *Clostridioides Difficile,* methicillin-resistant *Staphylococcus aureus* (MRSA) [12], increases in hospital LOS [12], hospital readmission rates [13], surgical site infections [14], and mortality [15]. Despite these reports, there is limited information on outcomes for hospitalized patients with a PcnA label in the obstetric and neonatal populations.

In the unadjusted primary outcomes, hospitalized PcnA patients and their neonates had longer LOS than non-PcNa patients. For the secondary unadjusted maternal outcomes, total cost of care, days of antibiotic use, and PICC line use were significantly different than non-PcnA patients. However, when these secondary outcomes were adjusted for BMI, age, twin pregnancy, and tobacco use, the only differences between the groups were that PcnA patients had a shorter duration of antibiotic use than non-PcnA patients and that PcnA mothers were more likely to have a neonatal LOS > 48 h compared to neonates of non-PcnA patients.

Inadequate GBS prophylaxis results from giving antibiotics other than ampicillin, penicillin or cefazolin. Current American Academy of Pediatrics (AAP) guidelines recommend continued physical exam and vital signs for 36–48 h after birth to assess clinical stability [16]. PcnA patients are more likely to receive either clindamycin or vancomycin for GBS prophylaxis and subsequently their newborns do not receive adequate GBS prophylaxis. Therefore PcnA mothers often stay with their newborn for the recommended 36–48 h, increasing their LOS. However, the other factors BMI, age, twin pregnancy, and tobacco use were enough of an influence on maternal LOS to shorten LOS to be no different than non-PcnA patients.

In the unadjusted outcomes, total cost of care was higher in PcnA mothers. The increase in costs may be related to the increased LOS and the increase in PICC use observed for PcnA mothers which could have added to the total cost of care in the unadjusted outcomes.

In the regression analyses that controlled for BMI, age, twin pregnancy, and tobacco use, neonates had an increased likelihood of a LOS > 48 h for those newborns born to a PcnA labeled mother. This is likely due to a 36–48 h observation period for those newborns with inadequate GBS prophylaxis, and a slightly longer LOS compared to routine newborn care. The duration of antibiotic treatment remained shorter for PcnA mothers in both the unadjusted and the adjusted outcomes for unknown reasons. We did not observe any differences in neonates born to PcnA labeled mothers or non-PcnA mothers in terms of transfer to a higher level of care, fetal death, or miscarriage. Although total cost of care was lower for non-PcnA patients compared to PcnA patients in the unadjusted outcomes, in the adjusted regression analyses, costs of care were similar between the groups. Several confounding factors that were not examined in this study may have contributed to the similarity in total cost of care such as existing comorbidities, other medications received in addition to antibiotics, or additional care unrelated to pregnancy in both the PcnA and non-PcnA groups.

Importantly, in this study, we did not see differences in maternal outcomes between PcnA labeled patients and non-PcnA labeled patients in terms of mortality, *Clostridioides difficile* infection, ICU admission, ED admission after discharge, or readmission. Possibly obstetric patients represented a healthier and younger population in general, especially when compared to the populations in other PcnA labeled studies [12, 13, 15].

This study had some limitations including that it was completed within a single healthcare system and may be constrained by regional geographic variations in underlying conditions or prevalence of a PcnA label. A power analysis was not done for this study, and further study with larger numbers of patients would be needed for conclusive outcomes.

Neonatal outcome data were limited and we were not able to include neonatal early-onset GBS infection as an outcome because neonates who required a transfer to a higher level of care were transferred to an adjacent pediatric hospital, after which data collection was not available. Therefore neonatal blood culture information was not included in the analyzed data set. Instead, transfer to a higher level of care was used as a surrogate marker for this outcome. Furthermore, the rate of infection for early onset GBS is about 0.23 cases per 1000 patients which would be difficult to measure accurately with our sample sizes [17]. Data were also not available on neonatal readmissions or visits to the ED.

Importantly, the approach to neonates of GBS positive mothers is changing. At the time of this analysis, the approach to GBS positive neonates was one of Categorical Risk Assessment or Enhanced Observation [16]. Categorical Risk Assessment included blood cultures and antibiotics for signs of clinical illness, clinical observation for 36–48 h for inadequate GBS prophylaxis, and routine neonatal care without clinical signs of illness and appropriate GBS prophylaxis. Enhanced Observation included blood cultures and empiric antibiotics for clinical illness, close observation for maternal illness or inadequate prophylaxis, and routine care for adequate prophylaxis without clinical illness.

Since the time of this analysis, a Neonatal Early-Onset Sepsis Calculator has been implemented [18]. This model begins with the probability of infection based on local and national rates of infection and uses a multivariate risk assessment to calculate an individual neonate’s risk of early onset sepsis [18]. Routine neonatal care, Enhanced Observation or blood culture and empiric antibiotic therapy are then recommended based on risk [16, 18]. This approach is likely to decrease LOS by increasing the number of neonates who can be cared for under routine care, despite inadequate GBS prophylaxis or maternal clinical illness, and lead to a decrease in the use of resources such as antibiotics and higher levels of care. In our study, we observed a longer LOS in neonates of PcnA labeled mothers which was a similar negative outcome to published reports describing the impact of a PcnA label.

## Conclusions

The American Academy of Pediatrics (AAP) recommends de-labeling of a penicillin allergy [16]. Following these recommended interventions could ensure that mothers and infants receive the recommended treatment of cefazolin or a penicillin for GBS prophylaxis. For future investigations in order to further assess the impact on a PcnA label on obstetric patients, a clinical trial could be performed in which pregnant mothers with PcnA are given specific Pcn testing to assess whether they have a true allergy versus adverse drug side effects or other confounding factors.

## Data Availability

The datasets used and/or analyzed during the current study are available from the corresponding author on reasonable request.

## Abbreviations

AAP: American Academy of Pediatrics
BMI: Body mass index
CI: Confidence intervals
CLABSI: Central line-associated bloodstream infection
ED: Emergency department
GBS: Group B streptococcus
IAP: Intrapartum antibiotic prophylaxis
ICU: Intensive care unit
IRB: Institutional Review Board
IRR: Incidence rate ratio
LOS: Length of stay
MRSA: Methicillin-resistant *Staphylococcus aureus*
PcnA: Penicillin allergy
PICC: Peripherally inserted central catheter
